# 

**Published:** 1922-12

**Authors:** 


### HOSPITAL and HEALTH

Established 1886 **REVIEW** No. 1857. Vol. LXXII.

New Series. Vol. II. No. 15 December 1922 Price Sixpence

CONTENTS.

| | PAGE |
|---|---|
| SPECIAL HOSPITAL APPEALS— | |
| Our Hospitals—To-day and To-morrow. By R. W. Harris and G. H. Hamilton. | 33 |
| Mass Contributions in London. By E. W. Morris, C.B.E. | 35 |
| The "New Movement" in Hospital Appeals. By Major J. R. Ainsworth-Davis, M.A., M.Sc. | 37 |
| Regular Contributions in South London. By R. J. Coles. | 39 |
| Hospital Co-operation in Westminster | 41 |
| NOTES AND COMMENTS— Tuberculosis in Domestic Animals—The Meeting of the Women's International Association—Outbreak of Small-pox in London—Music and Healing—Census Figures | 42 |
| Panelphobia and its Cure. By R. W. Harris. | 44 |
| National Health Insurance Notes and Comments | 45 |
| The Humour and Tragedy of Hospital Life. By Philip Inman. | 46 |
| Raising Money for Hospitals | 49 |
| Hospital Accounts and Finance. By Joseph E. Stone. | 50 |
| Hospital Finance | 52 |
| Hospital and Other News | 53 |
| Reviews | 54 |
| CORRESPONDENCE— Report of Committee of the Wilts Branch of the British Medical Association on County Medical Service—Nursing for the Insured—Co-operation in a Contributory Scheme | 57 |
| Recent Appointments | 59 |
| Government Publications | 59 |
| Hospital Needs and Hospital Appeals | 60 |

